# Social Network Analysis Reveals the Negative Effects of Attention-Deficit/Hyperactivity Disorder (ADHD) Symptoms on Friend-Based Student Networks

**DOI:** 10.1371/journal.pone.0142782

**Published:** 2015-11-12

**Authors:** Jun Won Kim, Bung-Nyun Kim, Johanna Inhyang Kim, Young Sik Lee, Kyung Joon Min, Hyun-Jin Kim, Jaewon Lee

**Affiliations:** 1 Division of Child and Adolescent Psychiatry, Department of Psychiatry, Seoul National University Hospital, Seoul, Republic of Korea; 2 Department of Psychiatry, Chung-Ang University College of Medicine, Seoul, Republic of Korea; 3 Division of Child and Adolescent Psychiatry, Department of Psychiatry and Institute of Behavioral Science in Medicine, Yonsei University College of Medicine, Seoul, Republic of Korea; 4 Korea Institute on Neuromodulation, EasyBrain Center, Seoul, Republic of Korea; Monash University, AUSTRALIA

## Abstract

**Introduction:**

Social network analysis has emerged as a promising tool in modern social psychology. This method can be used to examine friend-based social relationships in terms of network theory, with nodes representing individual students and ties representing relationships between students (e.g., friendships and kinships). Using social network analysis, we investigated whether greater severity of ADHD symptoms is correlated with weaker peer relationships among elementary school students.

**Methods:**

A total of 562 sixth-graders from two elementary schools (300 males) provided the names of their best friends (maximum 10 names). Their teachers rated each student’s ADHD symptoms using an ADHD rating scale.

**Results:**

The results showed that 10.2% of the students were at high risk for ADHD. Significant group differences were observed between the high-risk students and other students in two of the three network parameters (degree, centrality and closeness) used to assess friendship quality, with the high-risk group showing significantly lower values of degree and closeness compared to the other students. Moreover, negative correlations were found between the ADHD rating and two social network analysis parameters.

**Conclusion:**

Our findings suggest that the severity of ADHD symptoms is strongly correlated with the quality of social and interpersonal relationships in students with ADHD symptoms.

## Introduction

Attention-deficit/hyperactivity disorder (ADHD) is characterized by inattentiveness, hyperactivity, impulsivity, or a combination of these features. ADHD is one of the most common psychiatric disorders among children and adolescents [1]. A study conducted in the United States in 2004 with 3,082 8- to 15-year-olds found that 8.7% of the tested children had ADHD [2]. A Korean study by Cho et al. (2009) of 2,494 elementary school children found that 5.9% were diagnosed with ADHD and that 9.0% had ADHD tendencies but did not satisfy the full diagnostic criteria [3], suggesting that the prevalence of ADHD in South Korea is similar to that in the United States. ADHD symptoms in childhood and adolescence have negative consequences in multiple areas. Inattention or hyperactivity/impulsivity may cause significant impairment in academic, social, emotional, and familial functioning [4]. Previous studies indicate an increased risk of delinquency [5], substance use and abuse [6], and depressive or anxiety disorders [7]. If patients with ADHD are not properly treated, emotional problems emerge as maladaptive behaviors, and ADHD can evolve into social maladaptations and antisocial behaviors in adulthood [8]. Likewise, the difficulty in overall functioning associated with ADHD symptoms in childhood might persist [5].

Peer relationships and friendships are important to children’s social development [9]. Although peer-relationship difficulties are not a diagnostic criterion, children with ADHD are prone to social problems [10]. Studies on the social problems of children with ADHD show that most of these children have experienced peer rejection and have social-skill deficits [11, 12]. Difficulties in peer relationships among children with ADHD have been demonstrated in several previous studies. More than half of elementary school children with ADHD are “peer-rejected,” which is a large number compared to the 10%-15% of healthy children similarly categorized [13]. Additionally, 50–70% of children with ADHD experience peer-relationship problems [14]. Another study showed that 56–76% of children with ADHD fail to develop mutual friendships, a rate far higher than the 10–32% observed in healthy controls [10]. Childhood ADHD symptoms predict peer-relationship problems in adolescence [15] and increased internalization, resulting in poor social interactions in adulthood [16].

Peer-relationship problems, such as peer rejection and an absence of mutual friendship, are among the causes of poorer long-term outcomes in children with ADHD, despite adequate treatment [5]. The 14-month, NIMH-funded Multimodal Treatment of Attention Deficit Hyperactivity Disorder (MTA) study included psychosocial treatment for social-skill enhancement alongside the MTA medication algorithm, but no significant difference was seen between treatment groups [17]. Peer problems in children with ADHD do not respond well to standard ADHD treatment, such as medication, and present a major challenge to clinicians treating these children [18].

The aim of this study was to investigate the sociocultural backgrounds, mental-health problems and difficulties in peer relationships of children with ADHD symptoms to elucidate the correlation between ADHD symptoms and the quality of their social relationships. To this end, previous studies have used concepts such as peer rejection, peer neglect and social isolation. Another alternative concept is friendship nomination, which is widely used to identify mutual friendships. In this method, a child makes a list of close friends, and the close friends included in the list then make their own lists; children who nominate each other are considered mutual friends [13]. This technique can be used to assess dyadic peer relationships but is limited in terms of defining the social position of an individual or assessing complex dynamics of peer relationship within a group.

We used social network analysis to measure popularity, mediating roles (such as introducing and grouping between friends with awkward relationships), and social position within a group. We investigated the correlation between ADHD symptoms and social relationships using quantitative parameters rather than self-reports. To the best of our knowledge, there are no studies on the peer relationships of children with ADHD symptoms using social network analysis. The second purpose of this study was to elucidate the correlation between peer relationships and common comorbidities of ADHD, such as impulsiveness, learning disorders, and oppositional defiant disorder.

In summary, the aim of this study was to investigate the ADHD tendencies of community elementary school students and the association between these tendencies and impulsiveness, learning disorders, oppositional defiant disorder, and social-relationship difficulties to better understand the burden of ADHD in South Korea. We hypothesized that the measures of relationship quantity and quality calculated by social network analysis would decrease as ADHD symptoms and comorbidities increased in severity. This study analyzed the quality of peer relationships using a social-science technique known as “social network analysis,” a quantitative tool based on others’ assessments rather than self-reports.

## Methods

### 2.1. Participants

We selected schools with attention to the differences in school cultures between the metropolitan area and rural areas, as a school with strong regional characteristics might not be representative of the general population. We chose the Chungcheongnam-do district for reasons of accessibility and the city of Gongju for its high educational standards. There, we chose the two elementary schools with students of similar socioeconomic status that expressed the most interest in participation. Detailed information about the study was given to parents and children, and then written informed consent was obtained from parents, children, and homeroom teachers before the study entry. The study protocol was approved by the institutional review board of the Gongju National Hospital.

To ensure a sufficient number of participants, two schools were chosen, and the investigation was conducted simultaneously among sixth-graders at both schools. Sixth-graders were chosen because these students had attended school for the longest duration and therefore had time to establish relationships with other students. Additionally, these students were old enough to comprehend the content of the screening tools. There were no participants with suspected intellectual disabilities at either school, and there were no missing data in the questionnaires due to the active cooperation of the homeroom teachers, which made it possible to enroll the entire sixth-grade class in both schools. A total of 307 sixth-grade students were sampled from School A, and 255 were sampled from School B. The mean age was not significantly different between the two schools (A/B: 11.30±0.46/11.32±0.86, p = 0.70, Cohen’s d = 0.03). School A had slightly more students, but the gender ratios of the two schools were similar (A: 51.8% male, 48.2% female; B: 55.3% male, 44.7% female).

### 2.2. Instruments

Basic demographic questionnaires and the ADHD screening test were given to all participants. Data were obtained from homeroom teachers, who were likely to have the most information about the students. Students completed self-rated questionnaires with questions about their families, religion, and other social relationships, as well as clinical scales to assess impulsiveness, depression, and internet use. The homeroom teacher completed the scales used to assess ADHD, learning disorders and oppositional defiant disorder for each student.

This study used five self-rated scales and three teacher-rated scales. The self-rated scales included the Korean version of the Barratt Impulsiveness Scale (BIS) [19], the Korean version of the Children’s Depression Inventory (CDI) [20], the School Bullying Self-Rating Questionnaire (SBSRQ) [21], the Conners-Wells’ Adolescent Self-Report Scale (Short Form) [CASS(S)] [22], and six of the 10 items suitable for adolescents from the modified Lubben Social Network Scale (LSNS) [23].

The teacher-rated screening tools included the Korean parent and teacher ADHD Rating Scale (K-ARS) [24], the eight-item Disruptive Behavior Disorder Scale (DBDS) based on the DSM-IV to assess oppositional defiant disorder [25], and the Korean version of the Learning Disability Evaluation Scale (K-LDES) [26].

#### 2.2.1. Barratt Impulsiveness Scale-11 (BIS-11)

The BIS-11 is a self-report questionnaire designed by Patton (1995) to assess impulsivity and is composed of three subcategories, including six attention-impulsiveness items, eight motor-impulsiveness items, and nine non-planning-impulsiveness items. This questionnaire uses a four-point Likert scale to measure the frequency of each item (0 = rarely/never to 3 = almost), and the total score is the sum of the 23 items (range, 0–69). Cognitive impulsiveness assesses the tendency to respond or make decisions without deep thought, motor impulsiveness assesses the lack of inhibition of spontaneous behaviors, and non-planning impulsiveness assesses the tendency towards non-planning and non-consideration of safety issues before acting [19].

#### 2.2.2. Children’s Depression Inventory (CDI)

The CDI is a modified version of the Beck Depression Inventory developed by Kovacs (1983) to assess depressive symptoms in children. It includes 27 items that assess the mood status of a child for the past two weeks using a self-report form. Each item is graded on a scale of 0–2, and the total score ranges from 0 to 54 [20].

#### 2.2.3. School Bullying Self-Rating Questionnaire (SBSRQ)

The SBSRQ was developed by Kim (2007) to assess school bullying using a self-report questionnaire. The questionnaire contains 12 questions related to one’s experience of harassment and close peer relationships and is used to assess group bullying at school. All items are rated on a four-point Likert scale from 1 (not at all) to 4 (strongly agree), and the total score ranges from 12 to 48, with a higher total score indicating greater severity of being the victim of school bullying [21].

#### 2.2.4. Conners-Wells’ Adolescent Self-Report Scale (Short Form) [CASS(S)]

CASS(S) is used to assess ADHD symptoms in adolescent students. Twenty-seven items are graded on a scale from 1 to 4, and the total score ranges from 27 to 108, with a higher total score indicating a higher risk of ADHD. This measure consists of four subscales, including conduct problems (six items), cognitive problems (six items), hyperactive-impulsive (six items), and ADHD index (12 items) scales [22].

#### 2.2.5. Lubben Social Network Scale (LSNS)

The LSNS is a self-report questionnaire developed by Lubben (1988). It measures five aspects of social networks: family network, friendship network, helping others, confidant relationships, and living arrangements. Ten items are graded on a scale from 0 to 5, and the total score ranges from 0 to 50, with a higher total score indicating higher social support. This study used only the six items regarding family and peers that are appropriate for adolescents [23].

#### 2.2.6. Korean parent and teacher ADHD Rating Scale (K-ARS)

K-SARS was developed by DuPaul (1991) to evaluate ADHD symptoms in school-age children. It consists of 18 items based on the DSM-IV ADHD diagnostic criteria. Each item is rated on a four-point Likert scale of 0 (never) to 3 (almost every day) according to the frequency of the problem behavior. The sum of scores of odd-numbered items represents inattentive symptoms, and the sum of scores of even-numbered items reflects hyperactive-impulsive symptoms, with nine items in each category. The total score ranges from 0 to 54. In this study, the teacher-rated form was used to increase reliability [24].

#### 2.2.7. Disruptive Behavior Disorder Scale (DBDS)

The DBDS was designed for a caretaker to evaluate a child’s behavior based on the DSM-IV ADHD, oppositional defiant disorder (ODD), and conduct disorder (CD) diagnostic criteria. It includes 18 ADHD items, 8 ODD items, and 15 CD items, for a total of 41 items. In this study, we used only the 23 items assessing ODD and CD, and each item is rated on a four-point Likert scale of 0 (never) to 3 (almost every day). The total score ranges from 0 to 69 [25].

#### 2.2.8. Learning Disability Evaluation Scale (K-LDES)

The K-LDES is an 88-item questionnaire developed by McCarney (1983). It was translated and standardized in Korean in 1998. The seven standardized subscales (listening, thinking, speaking, reading, writing, spelling, and mathematical calculation) provide education-related assessments for learning disorders. The Learning Quotient (LQ) is the score of the sum of the seven subscales, adjusted to fit an average of 100 and a standard deviation of 15 [26].

### 2.3. Social network analysis of peer relationships

Social network analysis was developed in 1967 based on Stanley Milgram’s finding that “in American society, two strangers can be connected by an average of 5.2 acquaintances.” This method measured societal relationships not by subjective judgments but by mathematical equations [27]. Social network analysis, a promising method in social science, can be used to identify the central figure and outsiders of a group and to categorize an individual’s influence on his or her social circle [28]. Thus, social network analysis has emerged as a key technique in modern sociology and is now commonly available as a consumer tool. Social network analysis views social relationship in terms of network theory, with nodes and ties representing individuals and relationship between individuals, respectively.

A peer relationship network was constructed using the survey question, “Write down the names of 10 friends in the sixth grade of this school whom you have met outside of class, whose home you have visited or whom you frequently call on the phone.” In the social network analysis, among the many variables available, we selected the three that were most likely to represent peer relationships. The first variable was “degree,” which corresponds to how many other students selected the individual as a close friend. This variable is similar to “in-degree,” which is commonly used in social network analysis to show the degree of connection from outside. While “out-degree” is used to represent the number of connections going out from the individual, “in-degree” indicates how many friends regard that student as a close friend. The second variable was “centrality,” also called “betweenness centrality.” This variable calculates the possibility that when A and B are connected via the shortest distance possible, C exists somewhere along the path. Here, this variable indicates how often a student connected two other students in the friendship network. The third variable was “closeness,” which is decided by the total theoretical distance to all other nodes. This variable is similar to “in-closeness,” which is commonly used in social network analysis and summarizes the inverse of all of the lengths from all students to a particular student. Here, “in-closeness” indicates how closely a student is connected to all other students in the friendship network. A high “closeness” score indicates that the student has broad peer relationships, while a low score indicates the opposite [29]. Another advantage of social network analysis is that it can represent all relationships in the network using a map. The most common method of network mapping uses the Kamada-Kawai layout algorithm [30]. Members who are popular and have a larger number of relationships are located in the center, whereas those who have few relationships are located on the periphery.

The social network analysis tools Pajek version 2.04 (Vladimir Batagelj and Andrej Mrvar, http://pajek.imfm.si/doku.php?id=pajek) and Netminer version 4.0 (Cyram, South Korea, http://www.netminer.com) were used.

### 2.4. Statistical analyses

All statistical analyses were conducted using Predictive Analytics Software ver. 18.0 for Windows (SPSS Inc., Chicago, IL, USA) with a significance level of 0.05. A total of 562 participants were analyzed. The N was large enough for a normal distribution, so parametric methods were used. The children were divided using the K-ARS scores assessed by homeroom teachers into a normal group and a high-risk group. The cutoff score was 17, which is the score generally used in clinical settings [31]. The demographic data and clinical-scale scores were compared using independent-samples t-tests and chi-square tests. Pearson’s correlations were used to analyze the associations between the nine clinical-scale scores and the three social-network variables.

## Results

### 3.1. Comparisons of peer relationships and clinical-scale scores according to gender

The teacher-rated K-ARS score was significantly higher among male students. Male students also scored significantly higher than female students on the DBDS and the K-LDES. Female students scored significantly higher than male students on the CDI, suggesting a higher prevalence of depressive symptoms among female students than among male students. No significant differences were observed between male and female students with regard to degree, centrality and closeness (Table 1).

**Table 1:** Comparisons of peer relationship and clinical scale scores according to gender.

	Boy	Girl	t	p	Cohen’s d
K-ARS	7.10±8.94	2.27±5.31	7.88	<0.001**	0.66
BIS	26.42±8.55	26.48±8.63	-0.08	0.939	0.01
CDI	9.46±7.12	11.05±7.32	-2.59	0.010*	0.22
CASS(S)	16.34±9.72	14.79±9.61	1.91	0.057	0.16
LSNS	16.75±6.37	17.79±5.86	-2.02	0.044*	0.17
SBSRQ	3.16±4.47	2.79±4.13	1.03	0.302	0.09
DBDS	2.23±3.53	1.17±2.82	3.95	<0.001**	0.33
K-LDES	2.65±5.58	1.44±4.01	2.97	0.003**	0.25
Degree	7.72±4.65	7.63±3.91	0.27	0.788	0.02
Centrality * 100	0.24±0.25	0.25±0.31	-0.63	0.531	0.05
Closeness	0.13±0.03	0.13±0.03	-0.01	0.995	0.01

p-values are indicated as follows:

* p ≤ .05

** p ≤ 0.01.

K-ARS, The Korean ADHD Rating Scale; BIS, the Korean version of the Barratt Impulsiveness Scale; CDI, the Korean version of the Children’s Depression Inventory; CASS(S), Conners-Wells’ Adolescent Self-Report Scale (Short Form); LSNS, Lubben Social Network Scale; SBSRQ, School Bullying Self Rating Questionnaire; DBDS, Disruptive Behavior Disorder Scale according to DSM-IV; K-LDES, the Korean version of the Learning Disability Evaluation Scale; Degree, In-Degree; Centrality, Betweenness Centrality; Closeness, In-Closeness.

### 3.2. Comparisons of peer relationships and clinical-scale scores according to the severity and extent of ADHD symptoms

Fifty-seven children were included in the ADHD high-risk group, and 505 children were included in the normal group. The high-risk group scored significantly higher than the normal group on the CASS(S), DBDS, and K-LDES. The high-risk group had significantly lower LSNS, degree, and closeness scores than the normal group (Table 2).

**Table 2:** Comparisons of peer relationship and clinical scale scores according to the degree of ADHD symptoms.

	Normal	ADHD high-risk	t	p	Cohen’s d
BIS	26.25±8.49	27.84±9.12	-4.32	0.188	0.18
CDI	10.00±7.20	11.59±7.36	-1.57	0.118	0.22
CASS(S)	15.17±9.43	19.50±11.27	-3.20	0.001**	0.42
LSNS	17.46±6.01	15.76±6.83	2.00	0.046*	0.26
SBSRQ	2.82±4.11	4.18±5.54	-1.78	0.080	0.28
DBDS	1.23±2.62	6.19±4.67	-7.88	<0.001**	1.31
K-LDES	1.15±2.64	10.32±10.21	-6.75	<0.001**	1.23
Degree	7.84±4.25	6.53±4.68	2.20	0.028*	0.29
Centrality * 100	0.25±0.27	0.23±0.31	0.36	0.719	0.07
Closeness	0.13±0.03	0.12±0.04	2.12	0.038*	1.12

p-values are indicated as follows:

* p ≤ .05

** p ≤ 0.01.

K-ARS, the Korean ADHD Rating Scale; BIS, the Korean version of the Barratt Impulsiveness Scale; CDI, the Korean version of the Children’s Depression Inventory; CASS(S), Conners-Wells’ Adolescent Self-Report Scale (Short Form); LSNS, Lubben Social Network Scale; SBSRQ, School Bullying Self Rating Questionnaire; DBDS, Disruptive Behavior Disorder Scale according to DSM-IV; K-LDES, the Korean version of the Learning Disability Evaluation Scale; Degree, In-Degree; Centrality, Betweenness Centrality; Closeness, In-Closeness.

### 3.3. The correlations between peer relationships and the severity and extent of ADHD symptoms

**Fig 1:**
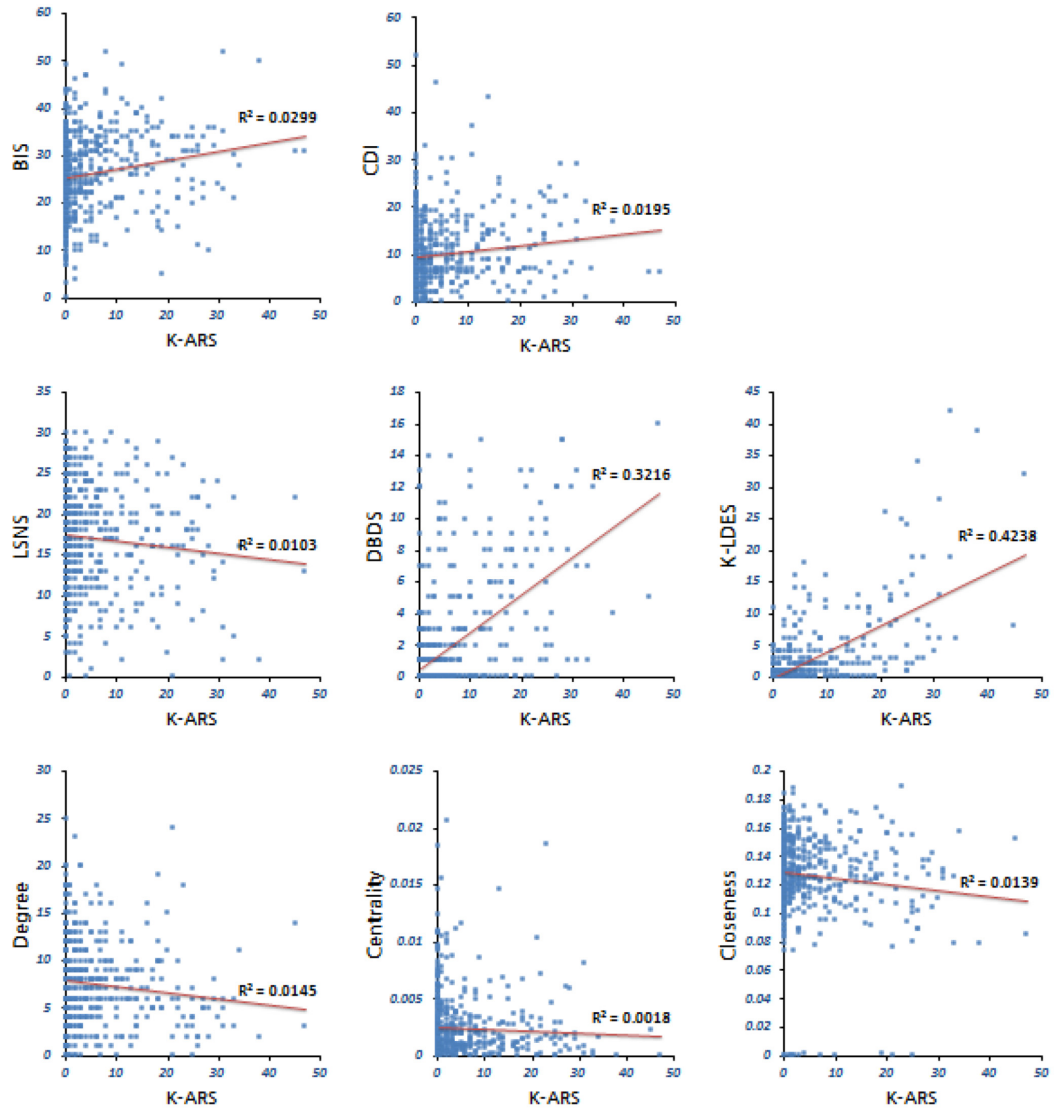
Scatter plots of the Pearson’s correlations between the K-ARS for teacher and the other clinical scales. K-ARS, The Korean ADHD Rating Scale; BIS, The Korean version of Barratt Impulsiveness Scale; CDI, The Korean version of Children’s Depression Inventory; CASS(S), Conners-Wells’ Adolescent Self-Report Scale(Short Form); LSNS, Lubben Social Network Scale; SBSRQ, School Bullying Self Rating Questionnaire; DBDS, Disruptive Behavior Disorder Scale according to DSM-IV; K-LDES, the Korean version of Learning Disability Evaluation Scale; Degree, In-Degree; Centrality, Betweenness Centrality; Closeness, In-Closeness

The severity and extent of ADHD symptoms were measured using the teacher-rated K-ARS. The K-ARS score was significantly correlated with the other 9 scales, including impulsiveness, depression, inattention-hyperactivity, social relationships, school bullying, oppositional defiant disorder, learning disorders, as well as decreased degree and closeness, but it was not correlated with centrality (Table 3). The scatterplot shows the correlation between the K-ARS scores and the other scale scores (Fig 1). Scores on the impulsivity, depression, oppositional defiant disorder, and learning disorder scales were positively correlated with the ADHD scores, whereas the social-relationship, degree and closeness data slant downward on the plots, demonstrating negative correlations (Fig 1). These findings suggest that the quality of social relationships, as well as the degree and closeness of peer relationships, deteriorate as ADHD symptoms become more severe.

**Table 3:** Correlation coefficients between K-ARS scores and other clinical scores (N = 562).

	K-ARS	BIS	CDI	CASS(S)	LSNS	SBSRQ	DBDS	K-LDES	Degree	Centrality	Closeness
K-ARS	1.000										
BIS	.173**	1.000									
CDI	.139**	.417**	1.000								
CASS(S)	.245**	.519**	.653**	1.000							
LSNS	-.101*	-.239**	-.210**	-.143**	1.000						
SBSRQ	.167**	.177**	.515**	.376**	-.144**	1.000					
DBDS	.567**	.000	.046	.080	.038	.091*	1.000				
K-LDES	.651**	.189**	.148**	.208**	-.203**	.137**	.243**	1.000			
Degree	-.120**	-.081	-.114**	-.072	.237**	-.169**	-.028	-.171**	1.000		
Centrality	-.042	-.069	-.111**	-.016	.183**	-.091*	.029	-.092*	.403**	1.000	
Closeness	-.118**	-.091*	-.167**	-.124**	.292**	-.263**	-.026	-.234**	.535**	.565**	1.000

Significant correlations in bold. p-values are indicated as follows:

*p ≤ .05

**p ≤ 0.01.

K-ARS, the Korean ADHD Rating Scale; BIS, the Korean version of the Barratt Impulsiveness Scale; CDI, the Korean version of the Children’s Depression Inventory; CASS(S), Conners-Wells’ Adolescent Self-Report Scale (Short Form); LSNS, Lubben Social Network Scale; SBSRQ, School Bullying Self Rating Questionnaire; DBDS, Disruptive Behavior Disorder Scale according to DSM-IV; K-LDES, the Korean version of the Learning Disability Evaluation Scale; Degree, In-Degree; Centrality, Betweenness Centrality; Closeness, In-Closeness.

### 3.4. Social network map analysis

**Fig 2:**
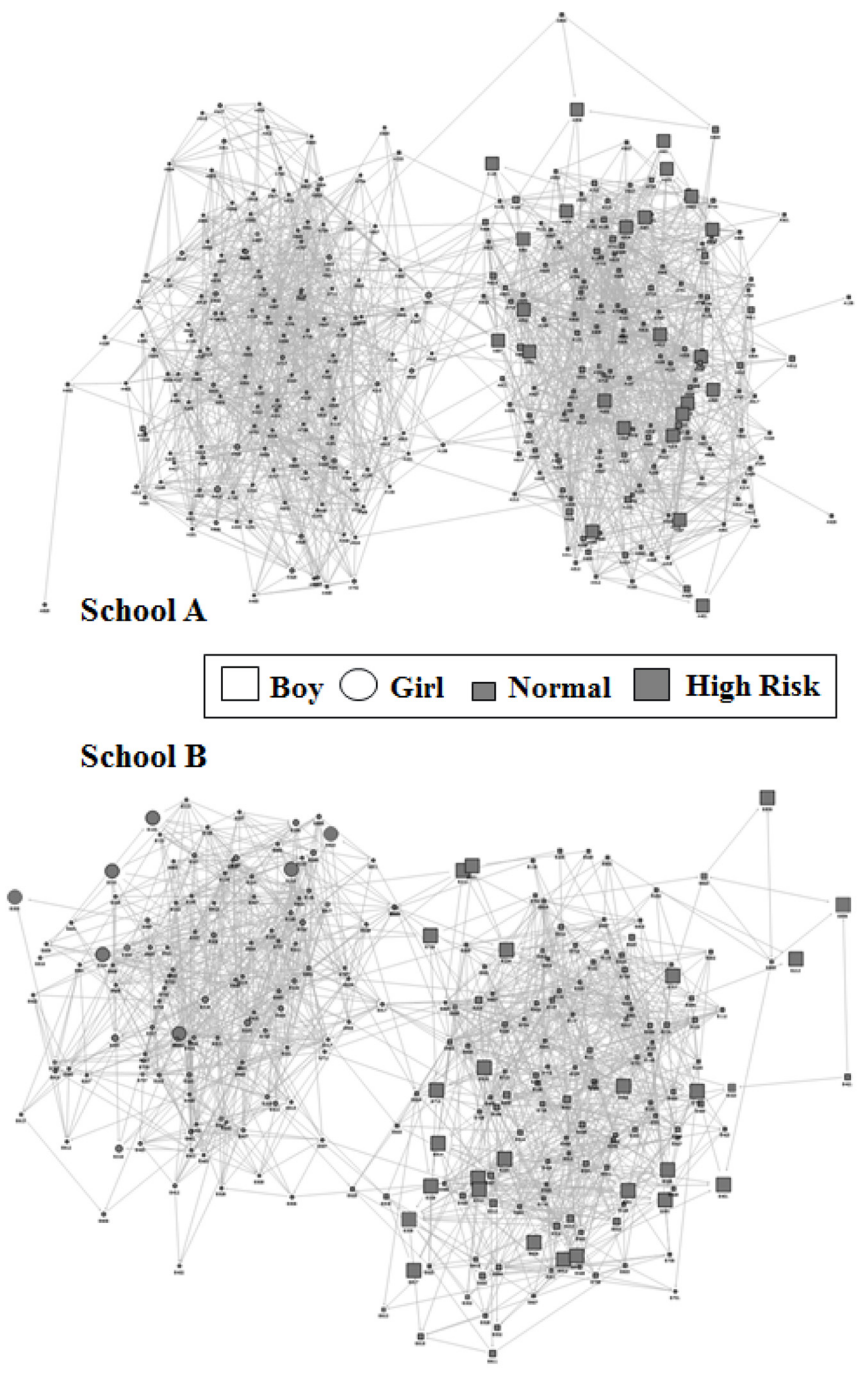
Social network analysis on peer relationships using the Kamada-Kawai layout algorithm. Boys are represented by squares, and Girls by circles and the large dots represent the ADHD high risk group.

The Kamada-Kawai layout algorithm can be used to present a network map to facilitate quick understanding of the peer relationships in a group [30]. In Fig 2, squares represent male students and circles represent female students. The large dots represent the high-risk group. The male and female relationship networks of both schools were somewhat separate, with only a few connections between the male and female groups. Only male students were classified into the ADHD high-risk group in School A. Likewise, male students constituted the majority of the ADHD high-risk group in School B. The high-risk group was located on the periphery in both schools (Fig 2).

## Discussion

The current study has several strengths. First, all sixth-grade students in two schools were included. If the elementary schools chosen in this study are representative of regional or national populations, then these data can be used to estimate the prevalence of ADHD or the high-risk population nationwide. However, these data might not be sufficient to generalize to the national population based on the sample characteristics. According to our results, Schools A and B have different proportions of students at high risk of ADHD and different demographic characteristics, all of which must be considered before the results can be used to draw more general conclusions. However, the schools are located in an urban area with high academic standards, where schools are prioritized. We therefore believe that these data are sufficiently representative of South Korea’s urban population. Second, we used teacher ratings, which might be more objective than ratings by parents [32]. Finally, we used social network analysis to assess the quality of peer relationships.

Our results are summarized below. In accordance with previous studies, we found that ADHD symptoms were more common in male students than in female students [33]. Furthermore, male students scored significantly higher than female students on the oppositional defiant disorder scale and the learning disorder scale, as expected, given that these conditions are more common among male students [34–36]. Female students scored significantly higher than males on the Lubben Social Network Scale, which assesses the frequency of contact with others. This finding suggests that females are more likely than males to call friends or family members. Finally, no significant gender differences were observed in the social network analysis. Previous studies have found differences in processing of peer relationships according to gender that have been suggested to result in differences in the development of boys and girls [37]. However, these previous studies assessed subjective perceptions of relationships with others [13], while this study quantified the complex dynamics of the groups using quantitative measures assessed by others.

Comparisons between the high-risk group and the normal group revealed characteristic differences in several areas. The high-risk group scored significantly higher than the normal group on the oppositional defiant disorder and learning disorder scales. These results align with those of previous studies reporting that people at high risk for ADHD are at increased risk for comorbid oppositional defiant disorder or learning disorders [38]. According to the social network analysis, the Lubben Social Network Scale score and the values of the degree and closeness variables were significantly lower for the high-risk group than for the normal group. Thus, high-risk students have weak peer relationships and are more likely to be outsiders in their class. A previous study showed that adolescents with ADHD symptoms are more likely to experience alienation among their peers and have difficulties forming meaningful relationships compared to those without ADHD symptoms [11]. One explanation for this finding is that youths with ADHD are more sensitive to stimulation from others and show more aggressive responses [11]. The higher rate of oppositional defiant disorder and inattentiveness-hyperactivity scale scores among the high-risk students in this study support this hypothesis.

The ADHD scale was significantly correlated with the other nine scales but not with centrality. This result implies that fewer people consider students with higher scores on the ADHD scale close friends, and they are at higher risk of being located at the periphery rather than in the center in peer relationships compared to other children. Interestingly, the correlation analysis also revealed that the in-degree and closeness scores in the social network analysis were significantly associated with five scales, including the ADHD scale, depression scale, social network scale, school bullying scale, and leaning disorder scale. These results suggest that students who have narrow relationships and are located at the periphery have higher rates of ADHD tendencies, experience more depressive symptoms, have limited relationships with their friends and family and are more likely to show perform poorly in school. However, because correlation analyses cannot detect causal relationships, it is not possible to determine whether or how these differences are causally related.

Finally, we generated a network map using the Kamada-Kawai layout algorithm, a common visualization method in social network analysis. This map shows separations in the peer connections between male and female students. Students at high risk for ADHD were concentrated among the males, and most were located at the periphery in both schools. These results are similar to those observed in the scale-scores analysis, again showing that the high-risk students were more likely than their peers to have weak peer relationships and to be treated distantly by other classmates. ADHD symptoms typically appear before age seven; however, behavioral problems more clearly manifest as children begin school. Therefore, ADHD is commonly diagnosed among school-aged children. Elementary school is an important period for peer relationship formation because students evaluate themselves through the eyes of their peers, who influence their forming self-image [39]. The formation of healthy friendships during this period predicts future personality and relationship development [40]. School is the most important social environment for school-aged children, where these children acquire social skills appropriate for their culture. Bullying in this period can cause serious negative effects on self-esteem and self-image and can lead to school maladaptation in the future [41]. The main symptoms of ADHD, including inattention and impulsivity, can be the cause of poor peer relationships low self-esteem, and mood symptoms, including depression and anxiety [42]. Peers interpret of the behavior of the children with ADHD as rude and aggressive, and eventually they gain a negative perspective and ignore the children with ADHD [43]. Previous studies have reported that children with ADHD are less popular and have more conflict with peers compared to typically developing children [13]. Children with ADHD develop a negative self-image [44], and this lowered self-esteem leads to lower values for degree and closeness, the measurement of the quality of social relationships in the social network analysis. All of the students who participated in this study were sixth-graders, which is the age at which children at high risk for ADHD are likely to experience alienation from their peers because of pre-existing ADHD symptoms. This experience of alienation can lead to poor school adaptation and cause peer problems. These students were located at the periphery of the group, as seen on the social-network map.

This study has several limitations. First, as previously mentioned, peer relationships, scores on mental-health screening tools, and the percentage of students at high risk for ADHD vary across sociocultural backgrounds. As a result, it is difficult to determine whether these results can be generalized to the South Korean population. Second, our participants were not patients but were students attending school, and the proportion of students classified into the ADHD high-risk group was relatively small (n = 57). This sample size limits the generalizability of our findings. Third, the current study was cross-sectional and did not use diagnostic tools with structured interviews. However, we used teacher-rated scales rather than self-report questionnaires or parent-rated scales to compensate for this limitation [45]. Finally, the study design rendered it impossible to determine causality (rather than correlation) between variables related to ADHD symptoms and peer relationships. Additional studies are needed to establish causal relationships.

Despite these limitations, we investigated mental-health problems among elementary school children using social network analysis to assess the association between ADHD tendencies and peer relationships. ADHD symptoms are highly related to impulsivity, depression, oppositional defiant disorder, learning disorders and the quality of peer relationships. In conclusion, screening for ADHD among young patients and intervening to actively manage and prevent peer problems might be helpful in enabling children to occupy a more central status among their peers and to grow into individuals who have broader relationships with others.

## Supporting Information

S1 Dataset The additional file ‘Supporting information files.zip’ contains data and analyzed result by SPSS. In this zip-compressed file, we provide two files: School_Data.xlsx; spss result_School.xls.
(ZIP)
